# How Did We End Up Using a Quadruple Guided Therapy Combining At Least Two Antibiotics with a PPI to Eradicate *Helicobacter pylori*?

**DOI:** 10.3390/jcm11175216

**Published:** 2022-09-03

**Authors:** Maxime Pichon, Julie Cremniter, Christophe Burucoa

**Affiliations:** 1 CHU Poitiers, Infectious Agents Department, Bacteriology Laboratory, 86021 Poitiers, France; 2 INSERM U1070 Pharmacology of Antimicrobial Agents and Antibiotic Resistance, University of Poitiers, 86073 Poitiers, France

Since the discovery of *Helicobacter pylori*, and even if the species is frequently susceptible to many antibiotics *in vitro*, only six of them (amoxicillin, clarithromycin, metronidazole, tetracycline, levofloxacin, and rifabutin) and bismuth salts could be considered as effective *in vivo* to eliminate *H. pylori* and have been used in recommended eradication treatments [1].

The therapeutic strategies to eradicate *H. pylori* were initially validated empirically, and from the first therapeutic trials, it appeared essential to combine several antibiotics with a proton pump inhibitor (PPI) to buffer the pH for efficacy, as gastric acidity denatures the antibiotics and prevents their activity *in situ* [2,3]. Nevertheless, as current data suggest that the use of PPIs to protect antibiotics from the catalytic action of gastric acidity is only partial, the restoration of efficacy cannot cover the entire nocturnal period [4]. It has recently been suggested that new molecules, such as vonoprazan, could successfully address this problem, without revising the complete data obtained so far [4]. It is therefore necessary to re-evaluate these antibiotics considering this new protective drug.

While monotherapy antibiotic trials have not given conclusive results (PPI-amoxicillin), multiple therapy trials (tetracycline, amoxicillin–metronidazole, or amoxicillin–clarithromycin), with or without bismuth salts, have not seemed any more conclusive [5,6]. In the early 1990s, these trials led to widespread recommendation of seven-days PPI–amoxicillin–clarithromycin empirical triple therapy. However, since the beginning of the 2000s, a decrease in eradication rates obtained with this triple therapy has been observed in many countries, mainly due to the increased prevalence of primary resistance to clarithromycin [7].

To adapt to the increasing prevalence of resistance, numerous clinical trials have demonstrated the efficacy of bismuth quadruple therapy or concomitant treatment with extended treatment durations of up to 14 days. Second- and third-line treatments using other antibiotics (levofloxacin, tetracycline, rifabutin, and metronidazole) have also been determined in clinical trials. 

Due to the high prevalence of resistance to antibiotics (mainly clarithromycin), since 2017, treatment has to be guided by the results of an antibiogram (or by PCR detecting mutations conferring resistance to clarithromycin) performed on gastric biopsy cultures [1]. Some industrial kits have also proposed the detection of some of these mutations in stool samples, with the objective to limit the invasiveness of the endoscopy (necessary to perform biopsy) [8]. However, here again, despite the assurance of antibiotic susceptibility, a combination of two effective antibiotics with PPI has remained necessary to obtain acceptable eradication rates approximating 95%.

All these therapeutic strategies (historical empirical treatments, recent empirical treatments taking into account resistance, second-line treatments, and guided treatments) could vary with regard to the choice of associated molecules, dosages, the duration of treatment, and the rate of administration. Few of these treatments have been previously studied *in vitro* or *in vivo* to obtain good pre-clinical data before moving on to clinical evaluations. For example, amoxicillin is used in all eradication treatments recommended as two-dose daily regimens, whereas for all other indications, it is now known that three to four doses daily are recommended [9]. One can also question the scientific and pharmacodynamic justification of the need to extend an antibiotic treatment up to 14 days, whereas for many other infections, treatment durations have been appreciably shortened in recent years.

In view of their worrying impact on the patient’s microbiome (leading to major clinical consequences, causing premature interruption of treatment), it is important to reflect on the interest of reducing the number of antibiotic treatments involved in eradication [10,11]. Indeed, the need to resort to combinations of two or even three antibiotics, with longer treatment durations, may be considered as demonstrating the partial ineffectiveness of the proposed treatments. It has also been observed that *H. pylori* infections of the gastric mucosa are not always due to a single strain (in case of “multiple infections”), which means that antibiotic combinations may be justified by the existence of minority subpopulations [8]. These minority populations maintain the need for proximally and endoscopically determining the antibiotic susceptibility testing profile of *H. pylori*. In addition, it is crucial to accurately determine the local concentration of antibiotic in the gastric mucosa, with the objective to optimize the antibiotic administration protocol. These data, with the use of PK/PD models, may help to optimize single antibiotic therapy, as well as determine the benefits and modalities of multiple antibiotic therapies, with an additive or synergistic effect, considering that no antagonistic effect can be observed in this situation.

Coupled with recent discoveries in molecular microbiology in case of the validation of the genotypic-phenotypic linkage, this information should lead to a re-evaluation of the antibiotic therapy administered to patients according to the bacteriological diagnosis [12]. New technologies which assess the presence of subpopulations resistant to first-line antibiotics at a distance from the site of infection, without a need to multiply the number of associations deleterious to the microbial ecology, could also be of interest to, and entrusted by, general practitioners in order to optimize the management of this infection, which can lead to a lethal outcome if not managed effectively [13,14,15].

## Data Availability

Not applicable.
